# Agreeing/Disagreeing in a Dialogue: Multimodal Patterns of Its Expression

**DOI:** 10.3389/fpsyg.2019.01373

**Published:** 2019-06-19

**Authors:** Laszlo Hunyadi

**Affiliations:** Department of General and Applied Linguistics, University of Debrecen, Debrecen, Hungary

**Keywords:** multimodality, communication, agreement, *Theme*, HuComTech

## Abstract

Multimodality is a key property of communication. Even though the main channel of exchanging information is textual, the written text also relies on paralinguistic means to convey additional information using various kinds of punctuation. Speech, too, is always present in contributing to the understanding of the wider scope of the context, represented by some restricted means, including typography, typesetting, and style. Gestures are also part of the text in its broader sense: public stage performances necessarily accompany the text, too. The complex of text, speech and gestures can be understood as a set of unified multimodal patterns that progress in time. This paper offers a comprehensive insight in the temporal structure of dialogue interaction through the study of the multimodal expression of agreement/disagreement in the HuComTech Corpus, presenting patterns of behavior discovered using the research environment *Theme*.

## 1. Introduction

Agreeing is not an autonomous state of mind of an individual: it is a behavioral event that necessarily involves an interaction requiring at least two actors and a subject. It comes about as a reflection on the truthfulness of some statement, view or opinion and can evolve under at least two conditions: (a) in the course of the interaction the actors realize that they share the same view independently from one another, or (b) one or more of the actors get convinced by the argument of the other actor(s). The process of agreeing takes different forms depending on these two different conditions: when the actors *A* and *B* share the same view independently, agreement by actor *B* usually follows a statement or elaboration by actor *A* as a backchannel of some sort (such as *Yes, indeed!*). When actor *B* gets convinced by actor *A* about the truthfulness of a given view, the act of agreeing by actor *B* may follow a question or some inquiry by actor *A* (such as *What do you think?* or *Do you agree?*), but other scenarios (such as those involving nonverbal events or pauses, virtually anything that prompts for a turn change) are also possible. Similarly to agreement, disagreement also evolves as a reaction act to a preceding prompt (Kakavá, 1993; Locher, 2004). Disagreement is often described as a behavior that reflects some kind of confrontation which, being understood as a function of face and politeness, should be avoided (cf. Sacks, 1973/1987; Brown and Levinson, 1978/1987; Leech, 1983; Pomerantz, 1984). Schiffrin (1984) shows, however, that it can also signal sociability, i.e., disagreement can even strengthen social relations. The role of context in the interpretation of these behaviors is widely recognized in pragmatics, even though the term itself is not sufficiently defined. Sifianou (2012) notes that, for proper interpretation, one even needs to consider longer periods of time allowing for the recognition of the development of personal traits and relational histories.

Agreeing and disagreeing are not in a simple binary relation: there can be several shades, degrees of this behavior (full or partial), indecision about what opinion to adhere to or advocate (uncertainty), or even a total lack of it (indifference). The recognition of these variants of agreeing/disagreeing is a key factor in conveying a successful conversation: not recognizing, or misinterpreting events of agreement can even lead to the total failure of the given interaction. Even though languages usually possess a number of lexical and syntactic means for the expression of this behavior, relying solely on the linguistic form may still be misleading. When, for example, actor *B* agrees with actor *A*, he/she would say “yes”; however, the same “yes” can also be used to suggest just the opposite, i.e., to mean disagreement—depending on the way “yes” is pronounced. Alternatively, one can agree or disagree by not even saying a word, just by keeping silent: again, it is the nonverbal behavior that contributes to the understanding of the context, effectively to the pragmatic interpretation of the event. Accordingly, in order to properly identify the instances of the pragmatic functions of agreement/disagreement, one has to consider all available modalities, both verbal and nonverbal, either audio or visual. However, there is one more challenge here. When someone expresses agreement by saying “yes” and nodding at the same time, this agreement is identified as the co-occurrence, the virtual temporal alignment of the two (verbal and gestural) events. But how can the wisdom of the proverb “silence gives consent” be justified, i.e., how can agreement be interpreted on the basis of the lack of the co-occurrence of any behavioral events? In fact, it is not the case that we face zero input here. We assume that we actually arrive at the interpretation of (some degree of) agreement after a certain period of observation, during which we collect pieces of data from all the available (verbal and nonverbal) modalities. In this process we go beyond just searching for simple temporal alignments of certain events, we rather try to identify behavioral patterns composed of events over a longer observation period. This is, in fact, a cognitive process in which the patterns identified in this way are matched against stereotypical patterns of behavior we are already aware of (either as innate or acquired ones), and the pragmatic function of the best match is assigned to the given pattern found in the observation period, in our case to the one associated with agreement/disagreement.

When designing the HuComTech Corpus, we wished to identify a variety of multimodal patterns of behavior across a given observation period. Using data from the resulted database, this paper has a focus on the discovery of temporal patterns related to agreement/disagreement. It describes the methodological basis of both building the corpus and analyzing and interpreting the data. Special emphasis is given to the research tool *Theme*: we both describe its theoretical foundations that facilitate the analysis of multimodal behavioral data and specify certain methodological questions of its application to the HuComTech Corpus. Finally, we present a selection of the most frequent temporal patterns associated with the pragmatic function of agreement discovered in the corpus and demonstrate their actual context in the recorded interactions.

## 2. The HuComTech Corpus: Its Structure and Annotation Scheme

The corpus is the result of joint efforts by researchers representing computational linguistics, pragmatics, engineering and information science and psychology. The project that started in 2009 aimed at a detailed study of human-human communication so as to offer important contribution to building various systems of human-machine interaction. From the outset it became clear that such a system should be multimodal, i.e., it should go beyond verbal communication and should include gestures as well that would enhance the user friendliness of such systems. It was also clear that the system should be capable of modeling a two-way communication. Namely, it should be able to be engaged in a recursive sequence of events of interaction by going beyond simply answering a query or fulfilling a request - it should “listen” to further reactions by the human user, evaluate them and act accordingly. Such a system requires two simultaneously active channels of communication, those of analysis and synthesis, through which the actors can continuously switch their roles as speaker and listener. The model we proposed as underlying our corpus building was designed to follow exactly this requirement (cf. Hunyadi, 2011). Naturalness of a two-way communication necessarily assumes that the actors are freely involved in the given topic and that the flow of interactions allows for unconstrained expression of gestures and emotions. Accordingly, we designed two kinds of dialogues: a predominantly formal one – in the form of a job interview with a set of predefined turns and a second, representing an informal conversation (for better data management the latter also followed some guidance, but allowed for individual variation). In order to better understand the possible structure of sequences of interactions and offer useful generalizations, the experimental scheme put emphasis on the following: turn management, the variation of intentions and the generation of emotions. The video recordings were made with 111 participants as speakers (60 male and 51 female, aged between 21 and 30) and two participants as agents (one male and one female, aged 25 and 28, respectively) with an average duration of the formal interviews being about 10 minutes and the informal conversations about 20 minutes. The resulted corpus has a total duration of about 50 hours (for further descriptive details of the corpus cf. Pápay et al., 2011; Hunyadi et al., 2012).

Even though our corpus building followed the main characteristics of multimedia corpus annotation in general, especially the DiAMSL framework (cf. Bach and Robert, 1979; Sperber and Wilson, 1986; Wilson and Sperber, 2002; Bunt et al., 2010), it differed from them in that in addition to assigning certain annotation labels as a result of multimodal observation, we also relied on unimodal observation. Whereas in multimodal observation all available modalities (in general, both video and audio) were involved, the unimodal observation followed either the video or the audio of the given recoding only. The rationale for this additional unimodal observation was that we wished to better identify which of the modalities in question has or have a specific contribution to the perception of certain communicative/pragmatic functions, including intentions, and emotions. By doing so we hoped to get a better understanding and a more detailed description of the individual differences among speakers in the expression of such functions as perceived and interpreted by the observers. Labeling of both verbal and especially nonverbal events for behavioral categories followed an elaborate protocol during which the annotators were continuously involved in discussions. The specificity of between-subject differences was captured by a significant period of familiarizing with the overall behavior of each of the subjects on the recording before the actual annotation could start.

There were 40 levels of annotation, including video and audio; either multimodal or unimodal, representing either physically measurable events or non-physical, abstract ones, the latter as resulting from pragmatic interpretation (for the development of the pragmatic features of the corpus cf. Németh, 2011). Each level of annotation was done independently from any other annotation. Each file was annotated by one annotator but checked by another one. Inter-annotator agreement was assured by frequent consultations and debates. The annotation of physical events was naturally all unimodal, since they came from direct visual or audio observation or measurement, including, based on video, the direction of gaze, blinking, hand- and head movements, posture and, based on audio, pitch movements, changes of intensity, silence, overlapping speech, start, and end of speaking. Emotions were annotated in three ways: multimodally (observing both video and audio) and unimodally, following audio, and again unimodally, following video (facial expressions). As a matter of fact, these three different modalities of observation effectively showed differences in the scope and intensity of emotions observed across the modalities for one and the same recording. In addition, this approach, as probably expected, also gives the chance to capture the speaker-specificity of the expression of emotions.

With these ideas in mind, an extensive annotation was performed on all the 222 recordings. The texts of the speakers and their agents made up about half a million running words, and each of the words was time-alined within the given speech flow, an especially helpful feature of the corpus to associate non-verbal events with the corresponding spoken text. The text was also annotated for morphology and syntax, another important feature which, due to its information about linguistic flaws and incompleteness during a conversation can contribute to both learning more about the cognitive flow of linguistic behavior and to building more natural interactive systems. Video annotation included the classes of facial expression, gaze, eyebrows, headshift, handshape, touchmotion, posture, deixis, emotions and emblem. Audio was annotated for the classes of intonation phrase, emotions and discourse, in addition to phonetic events within speech, such as silence, hesitation, restart, non-phonemic sounds, and noise. Automatic methods were applied to the annotation of the phonetic features of the sound track: in addition to marking the absolute values for F0 and intensity, a special algorithm (Szekrényes, 2014, 2015) was used to annotate stylized intonation and intensity contours of speech in order to capture the contribution of speech prosody to the multimodal expression of the pragmatic content of the interaction. The pragmatic levels of annotation included the classes of turn management, attention, agreement, deixis and information structure. Since each and every event of any class was annotated as time-alined, the scheme made it possible to associate virtually any event of any kind with another event. Due to computational restrictions (of which we'll be more specific in the next section) this work is concerned with only the following classes: the physical classes of gaze, handshape, headshift and posture, and the interpretive pragmatic classes of agreement and emblem. The present study uses data from this restricted set of classes from the HuComTech Corpus to identify multimodal patterns associated with the behavioral events of agreement/disagreement.

## 3. *Theme*: Why This Computing Environment Can be Suitable for Identifying Patterns of Behavior From the HuComTech Corpus

One faces at least four challenges when attempting to discover multimodal patterns of behavior: (a) even though there are stereotypical assumptions about what modalities or events can be associated with a given pragmatic function in behavior, the set of such candidates may not eventually be a closed one; (b) even if just considering a stereotypical set of events making the pattern for the given behavior, it is often the case that one or another event can be missing from the pattern without violating the given functional interpretation, that is, some (or sometimes all?) of the events in a stereotypical description of a pattern can be optional; (3) whereas the constituting events can either co-occur with or follow one another, their temporal sequence does not necessarily follow adjacency, i.e., one or more events can occur between two “stereotypical” events as “noise”; (d) even though behavioral patterns occur in time, the temporal sequence of the constituting events cannot be determined as having a constant, stable discrete duration, rather, the interval between two events within a pattern can only be determined by statistical probability. *Theme* (Casarrubea et al., 2015, 2018; Magnusson et al., 2016; patternvision.com) appears to capture the optionality of possible pattern-making events, overcome the strict adjacency requirement of certain analyses and surpass the constraint of predetermined intervals between events as presupposed by time series analysis. As such, it captures the inherent property of behavioral patterns of (between-subject and within-subject) variability both in composition and timing and define occurrences of patterns by statistical probabilities. *Theme* is a statistical environment that computes all these conditions and determines which of the theoretically possible co-occurrences or sequences of any two events make a minimal (i.e., first level) pattern. Computation by *Theme* is based on the concept of critical interval: it determines which of the temporal occurrences of any two events, such as A and B are within an interval that satisfies the condition of a certain probability, say *p* = 0.005. *Theme* recursively associates any two events into a minimal pattern, or minimal patterns into more complex patterns, thus building a theoretically non-finite hierarchy of events and patterns. *Theme* has one more important concept: whereas, intuitively, one associates an event with its duration, *Theme* considers both the starting point and the ending point of such an event as a separate event, and it associates them with any other event individually to form a pattern. This is how *Theme* can capture the difference between the following two situations: in the first, *B* starts answering *A*'s question while *A* is still speaking, in the second, *B* only starts answering after *A* has finished the question. The fact that *Theme* is fundamentally based on discrete points of time associated with any kinds of events, allows us to attempt to discover even behavioral patterns which are hidden from the naked eye, i.e., without relying on stereotypes. The only restriction for *Theme* is, however, understandable: it can only identify patterns based on events that were previously annotated. Theoretically, the responsibility of the selection of categories (classes in *Theme*'s terms) to be annotated solely lies on the design of the annotation scheme. Our work, however, is also restricted by the computational power available at present: in order to successfully manage a reasonable amount of computation, our search for patterns was restricted to the classes of annotation enumerated in the previous section. We hope, however, that the resulting patterns will prove to be representative of agreement and they will correspond to our everyday intuitions. This is what the next section is intended to offer.

## 4. Patterns of Agreement As Discovered by *Theme*

**Figure 1:**
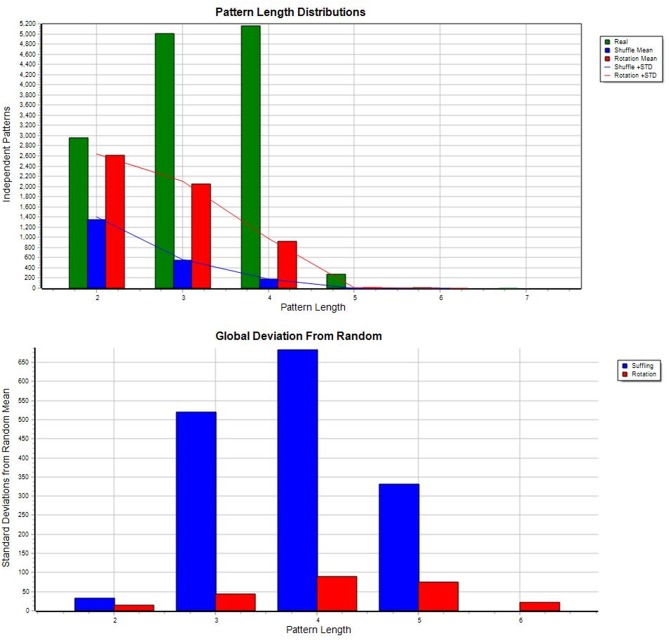
Real vs. random (Upper) and devition from random (Below) using file: i082.

All analyses were done using *Theme 6* (full version) on a virtual machine equipped with 64 GB memory and 20 virtual CPUs. Due to the large amount of data to process the corpus was divided into 10 smaller chunks, and the same procedure was followed for each of them. The data files contained all the annotations available in the corpus, including those which were not targeted in the current research. For pattern discovery, classes and event types relevant to this study were only selected using the Reorganize Data option. The basic search settings were as follows: significance level: 0.005, minimal occurrence of candidates for a pattern: 3, univariate patterns: no, minimum d1: 1, maximum d1: 1500 (i.e., the critical interval between any two events to be considered as candidates for a pattern fell between 1 and 1500 ms), maximum number of search levels: 3, exclude frequent event types: 0.25, top percent kept per level: 75. The values of the three latter parameters reflect the existing computational restrictions imposed on the amount of the data: even though there was a chance to discover even more complex patterns searching at levels higher than the preset 3, even the available 64 GB memory would not have been sufficient for that. The reduced values of the other two parameters reflect the same computational constraint, too. The data were randomized using shuffling and rotation (an outstanding testing feature of *Theme* to ensure that the patterns identified are indeed generated within some specific temporal structure of events). Figure 1 allows us to compare the number of patterns derived from either real or randomized data: from level 2 and upwards (with 3 or more pattern constituents) patterns tend to be specific to real data, and as such, to the communicative situations the real data are based on:

Before turning to the actual analysis of patterns of agreement, let us have a look at some basic data about the corpus as a whole.

The total set of 111 formal and 111 informal dialogues (each by the same 60 male and 51 female speakers) was included in the discovery of patterns. Annotations of an action, such as “head turn to the right based on video observation” were separated into two events: one for the beginning, one for the end of the given head turn, such as “v_head,b,right” and “v_head,e,right.” Out of the total 3,929,630 events annotated (1,964,815 for the beginning and the same amount, 1,964,815 for the end of an action) a large number of them were actually found in one or another pattern: 1,699,825 denoted the beginning (86.5% of all annotated “begin” events), and 1,469,751 denoted the end of a given event (74.8% of all annotation “end” events). The events contributed to the complexity of the resulting patterns in such a way that more than one instance of one and the same event type could be part of a given (more complex) pattern, as shown in file f003:


   ((( v_head,b,right  v_head,e,right )
        v_head,b,right )  v_head,e,right ) 


This pattern tells us that the speaker turned his head to the right twice (“v_head” = head movement annotated using video observation, “b” = begin, “e” = end, “right” = head movement to the right). The dependency denoted by the bracketing also showed that the first head movement was shorter than the second one. In all, the occurrence of the “begin” events in patterns was about 16% higher than that of the “end” events showing that the beginning of an action was somewhat more specific to a pattern than its end.

The total number of pattern occurrences (i.e., pattern tokens) throughout the corpus was 967,446. As for their distribution by gender, male speakers showed more patterns than females: males: 599,533, females: 367,913. As for their distribution by formal/informal dialogue type, the informal dialogues had 37.9% more patterns than would be expected by the difference by duration between formal and informal dialogues (257,478 vs. 709,968 patterns in about 10 vs. 20 min dialogues, respectively).

Search for patterns by *Theme* is done recursively. It means that once a pattern is discovered at search level 1, this pattern is carried on to the next level where *Theme* attempts to associate it with another event or another pattern to make it part of a more complex pattern. In this process *Theme* may find that pattern 1 found at level 1 will be associated with pattern 2 found at level 2 in a resulting pattern 3 so that pattern 1 is higher on the hierarchy that pattern 2 – this is the case of forward embedding; or, alternatively, pattern 2 can be higher in the hierarchy than pattern 1 in the resulting pattern 3– the case of backward embedding. As for the whole corpus we find that out of the 967 446 pattern occurrences 82.9% (802 236) have some sort of embedding (i.e., they are generated at level 2 or level 3), evenly distributed as forward or backward embeddings: single forward embedding: 291,771, single backward embedding: 289 663, double forward embedding: 108,893, double backward embedding: 111 909. These data demonstrate how important it is in the modeling of the generation and perception of behavioral patterns to consider a pattern as a temporal complex which is not necessarily built as just a forward looking temporal sequence of events, but where some events can have a backward structural reference to one or more temporally preceding events as well.

Let us now turn to the events and patterns associated with the expression of the pragmatic function of agreement.

The following video classes were included in the discovery of associated patterns: the physical descriptive classes of v_gaze, v_hand, v_head and v_posture (the prefix “v_” in each case representing video observation), and the interpretive class of v_embl (=emblem; including various shades of agreement). Table 1 shows the occurrences of items representing the emblem class:

**Table 1:** Emblem class: frequency of items in annotation and in patterns.

**Item**	**Annotation entries # with item**	**# Files with item**	**Patterns # with item**	**Files with item # in patterns**	**Type-token ratio files with items in annotations vs. files with items in patterns**
Attention	5154	66	6707	13	76.85
Default disagree	5136	186	7349	111	69.89
Agree	4798	141	7091	71	67.66
Doubt	1452	91	645	23	225.12
Block disagree	1196	109	1363	47	87.75
Refusal	594	65	196	6	303.06
More-or-less	516	70	0		n/a
Doubt-shrug	342	51	405	5	84.44
Surprise-hands	290	45	68	3	426.47

We can see that the frequency of items in annotations does not necessarily coincide with their frequency in actual patterns: *attention*, the most frequent annotation item is only ranked as the 3rd most frequent item occurring in a pattern, it is preceded in frequency by both *default disagree* and *agree*. These frequency data give us a glimpse of the nature of the conversations recorded: there were many moments of attention, as a natural component of a dialogue, but the fact that there were more moments of (default) disagreement than agreement also suggests that the interaction was fairly free. The small number of items *refusal, doubt-shrug* and *surprise-hands* suggests that the interaction did not include much of direct confrontation. An additional series of annotation to determine the degree of agreement revealed that there were 2331 instances of the item *uninterested* – yet another reflection of the speaker to some moment of the interaction. Its relatively small frequency shows again that the actors were duly engaged in the dialogues.

The following tables show a subset of patterns observed with the above items in the emblem class – the association of these items with events of the head, hand, and gaze classes:

Tables 2–4 above show that gaze movement was more frequently annotated than either head or hand, indicating again that there was much eye contact between the actors—as expected from an active interaction in general. This suggestion is also supported by the large number of nods as well as blinks, both of them usually accompanying conversational events. The speakers held their hand flat most of the time, suggesting again the non-confrontational nature of the dialogues; whereas the predominant use of the right hand against the left hand speaks about the predominant right-handedness of the actors.

**Table 2:** Emblem—frequency of items from the head class.

**Nod**	**Turn**	**Raise**	**Lower**	**Right**	**Left**	**All**
3683	1672	1869	1966	3946	2128	15264

**Table 3:** Emblem—frequency of items from the hand class.

	**Flat**	**Spread**	**Crossing fingers**	**Right**	**Left**	**Fist**	**Index-out**	**Broke**	**All**
v_hand	3283	461	166	4041	496	290	225	21	8983

**Table 4:** Emblem—frequency of items from the gaze class.

	**Blink**	**Up**	**Down**	**Right**	**Left**	**Forwards**	**All**
v_gaze	3801	3981	7658	3496	2156	13365	34457

Tables 5–9 show the role of gaze in the expression of various items (subcategories) associated with agreement (“b” and “e” denote the beginning and the end of an action, respectively):

**Table 5:** Emblem/agree—frequency of items from the gaze class.

	**blink**	**b, up**	**e, up**	**b, down**	**e, down**	**All**
b, agree	1186	110	7	897	1385	3585
e, agree	1225	140	19	841	654	2879

**Table 6:** Emblem/disagree—frequency of items from the gaze class.

	**Blink**	**b, up**	**e, up**	**b, down**	**e, down**	**All**
b, disagree	42	7	7	10	16	82
e, disagree	13	0	0	13	27	53

**Table 7:** Emblem/doubt—frequency of items from the gaze class.

	**Blink**	**b, up**	**e, up**	**b, down**	**e, down**	**All**
b, doubt	240	36	57	108	139	580
e, doubt	218	62	88	112	133	613

**Table 8:** Emblem/refusal—frequency of items from the gaze class.

	**Blink**	**b, up**	**e, up**	**b, down**	**e, down**	**All**
b, refusal	57	0	7	29	10	103
e, refusal	13	0	7	34	13	67

**Table 9:** Emblem/block—frequency of items from the gaze class.

	**Blink**	**b, up**	**e, up**	**b, down**	**e, down**	**All**
b, block	13	0	0	8	5	26
e, block	11	0	0	22	21	54

Among the many possible observations let us just notice the role of *blink*: it is mostly present as accompanying agreement, either its beginning or its end (even if with fewer occurrences, the same it true of *disagreement*). The beginning of the action of agreement is also strongly associated with “e,down,” i.e., the speaker stops gazing downwards—effectively looks up, most probably meets the eyes of the agent. When, also frequently, the speaker begins looking down (“b,down”) while starting agreeing, it may suggest a moment of deliberation and may eventually take his/her turn to continue the conversation.

Finally, let us have a closer look at the most frequent patters associated with agreement. In Tables 10–14, following *Theme*'s convention, each pattern is shown to consist of at least two event types. Event types making up a pattern are separated by a space. Each event type starts with the name of a given class, followed by “b” or “e” for “begin” or “end,” followed by the name of the item within the given class. As an example of this general syntax of notation cf. the first pattern in Table 10:


  ( up_agr,b,default_disagree up_agr,e,
        default_disagree )

event type 1: up_agr,b,default_disagree
  where
  “up_agr” = unimodal pragmatic class of agreement,
  “b” = the beginning of the event,
  “default_disagree” = an item of the class of agreement, i.e., an event of default disagreement.
event type 2: up_agr,e,default_disagree
  where
  “up_agr” = unimodal pragmatic class of agreement,
  “e” = the end of the event,
  “default_disagree” = an item of the class of agreement, i.e., an event of default disagreement.

**Table 10:** The most frequent patterns of agreement consisting of 2 events.

**Patstring**	**Number of files**	**Total number**
( up_agr,b,default_disagree up_agr,e,default_disagree )	90	490
( v_head,b,shake up_agr,b,default_disagree )	37	221
( v_head,e,shake up_agr,e,default_disagree )	32	182
( up_agr,e,default_disagree v_head,e,shake )	33	174
( up_agr,b,uninterested up_agr,e,uninterested )	25	158
( up_agr,e,default_disagree v_gaze,b,forwards )	33	157
( mp_spcommact,b,constat up_agr,b,default_disagree )	36	155
( up_agr,b,default_disagree v_head,b,shake )	32	143
( up_agr,b,default_disagree v_head,e,shake )	28	142
( up_agr,b,default_disagree v_gaze,b,forwards )	26	125

**Table 11:** The most frequent patterns of agreement consisting of 3 events.

**Patstring**	**Number of files**	**Total number**
( v_head,b,shake ( up_agr,b,default_disagree v_head,e,shake ))	7	44
( v_head,b,shake ( up_agr,b,default_disagree up_agr,e,default_disagree ))	10	41
( up_agr,b,default_disagree ( v_head,e,shake up_agr,e,default_disagree ))	8	41
(( v_head,b,shake up_agr,b,default_disagree ) v_head,e,shake )	6	35
( mp_spcommact,b,constat ( up_agr,b,default_disagree up_agr,e,default_disagree ))	10	34
(( mp_spsuppact,b,backch up_agr,b,default_disagree ) mp_spsuppact,e,backch )	8	29
( up_agr,b,default_disagree ( mp_spcommact,e,constat up_agr,e,default_disagree ))	9	28
(( up_agr,b,default_disagree up_agr,e,default_disagree ) v_gaze,b,forwards )	7	27
(( v_head,b,shake up_agr,b,default_disagree ) v_gaze,b,forwards )	3	26
( up_agr,b,default_disagree ( v_head,b,shake v_head,e,shake ))	7	25

**Table 12:** The most frequent patterns of agreement consisting of 4 events.

**Patstring**	**Number of files**	**Total number**
(((v_head,b,shakeup_agr,b,default_disagree) v_head,e,shake)up_agr,e,default_disagree )	4	16
((v_head,b,shakeup_agr,b,default_disagree ) (v_head,e,shakeup_agr,e,default_disagree))	4	15
(v_head,b,shake(up_agr,b,default_disagree (v_head,e,shake up_agr,e,default_disagree )))	3	12
(((v_head,b,shakeup_agr,b,default_disagree ) v_gaze,b,forwards)mp_spcommact,e,constat)	2	11
(v_gaze,b,forwards((v_gaze,e,forwardsv_gaze,b,down ) up_agr,e,default_disagree))	1	10
(v_head,b,shake(up_agr,e,default_disagree (v_head,e,shakev_gaze,e,forwards)))	1	10
(up_agr,b,default_disagree( v_head,b,shake (v_gaze,b,forwardsmp_agcommact,e,constat)))	1	9
((up_agr,b,default_disagree (v_gaze,b,down up_agr,e,default_disagree ))v_gaze,e,down )	1	9
(((v_gaze,e,forwardsv_gaze,b,down) up_agr,e,default_disagree )v_gaze,e,down )	1	9
(mp_spcommact,b,constat(up_agr,b,default_disagree (up_agr,e,default_disagree mp_spcommact,e,constat)))	3	9

**Table 13:** The most frequent patterns of agreement consisting of 5 events.

**Patstring**	**Number of files**	**Total number**
((( v_head,b,shake up_agr,b,default_disagree ) v_gaze,b,forwards ) ( up_turn,b,endsp up_att,b,paying ))	1	5
((( up_turn,b,endsp up_turn,b,intendsp ) ( mp_spcommact,b,constat up_agr,e,uninterested )) v_gaze,b,forwards )	1	4
(( v_head,b,shake up_agr,b,default_disagree ) (( v_head,e,shake up_agr,e,default_disagree ) v_gaze,e,forwards ))	1	4
((( v_head,b,shake up_agr,b,default_disagree ) v_gaze,b,forwards ) ( v_head,e,shake up_agr,e,default_disagree ))	1	4
( v_gaze,b,blink (( v_gaze,e,blink v_gaze,e,up ) ( up_agr,b,block_disagree up_agr,e,block_disagree )))	1	4
((( v_head,b,shake up_agr,b,default_disagree ) v_head,e,shake )( up_agr,e,default_disagree mp_agsuppact,b,backch ))	1	3
((( up_agr,b,default_disagree v_gaze,b,forwards ) ( up_agr,e,default_disagree mp_agsuppact,b,backch )) mp_spcommact,e,constat )	1	3
((( up_agr,b,default_disagree mp_spsuppact,b,backch ) ( v_head,e,shake up_agr,e,default_disagree )) v_gaze,b,forwards )	1	3
(( mp_spsuppact,b,backch up_agr,e,uninterested ) (( v_gaze,b,forwards mp_agcommact,b,constat ) mp_agsuppact,e,backch ))	1	3
( mp_spsuppact,b,backch (( up_agr,e,uninterested v_gaze,b,forwards ) mp_agcommact,b,constat mp_agcommact,e,constat )))	1	3

**Table 14:** The most frequent patterns of agreement consisting of 6 events.

**Patstring**	**Number of files**	**Total number**
((( v_head,b,shake up_agr,b,default_disagree ) v_head,e,shake )( up_agr,e,default_disagree ( mp_agsuppact,b,backch mp_spcommact,e,constat )))	1	3
((( mp_spcommact,e,constat mp_spcommact,b,constat ) v_gaze,b,down )(( up_agr,b,default_disagree mp_spinf,e,new ) mp_sptopic,b,t_elab ))	1	3
((( v_gaze,e,left v_gaze,e,forwards ) up_agr,e,default_disagree )( mp_agtopic,b,t_elab ( v_head,b,right v_head,e,lower )))	1	3
((( v_head,b,shake up_agr,b,default_disagree ) ( up_agr,e,default_disagree up_agr,b,default_disagree )) ( v_head,e,shake up_agr,e,default_disagree ))	1	3
(( up_agr,b,block_disagree ( mp_sptopic,b,t_init mp_sptopic,e,t_init )) (( mp_sptopic,e,t_elab mp_agcommact,b,constat ) v_hand,e,right,flat ))	1	3

One more note: the prefixes “v_” and “up_” equally stand for video observation. The difference is that “v_” stands for events definable by their physical form, such as direction, whereas “up_” stands for an event definable by its content, as the result of the interpretation of a pragmatic event that is based on video observation.

Table 10 confirms our stereotypes regarding disagreement: it is strongly associated with head shake in a number of files (recordings). Its temporal alignment with disagreement is also obvious: the start of disagreement aligns with the start of head shake, its end aligns with the end of headshake. It is also interesting to notice that starting looking forward can be associated with both the beginning and end of disagreement, the gaze direction suggesting some cognitive processes corresponding to the current status of disagreement. When the pattern has opposite values of begin/end of the two constituents as in ( up_agr,b,default_disagree v_head,e,shake ), it may suggest that the head shaking started earlier than disagreement could be observed—showing that it is also a possible variant for the pragmatic situation.

Table 11 confirms what we also saw in the simplest, two-event patterns: head shaking is found both as preceding and following the expression of disagreement. In addition, constatives and backchannel are also among the most frequent events taking part in more complex patterns, including embedding.

As shown above, head shaking is a main constituent both in the forward and backward embedding patterns. The intensive use of gaze movement by one speaker in the pattern ((( v_gaze,e,forwards v_gaze,b,down ) up_agr,e,default_disagree ) v_gaze,e,down ) suggests an active cognitive process during the course of disagreement (the sequence “ending looking forward + starting looking down” takes place while the speaker is disagreeing), and the speaker ends looking down right after finishing the act of disagreeing.

The set of the above, already longer patterns sheds light on the wider context of disagreeing, from just the intension of speaking to the start or end of speaking, backchannel and constative acts. Importantly, these data show that even these more complex patterns can have statistical probability.

Table 14 with its even larger context indicates the recursive role of hand shaking, backchannel and constative acts in building even loger behavioral patterns:

Finally, let us see the single longest, 7-item pattern of agreement:


  ((( mp_spcommact,e,constat mp_spcommact,b,
    constat )
  v_gaze,b,down )(( up_agr,b,default_disagree
    mp_spinf,e,new )
  ( mp_sptopic,b,t_elab mp_spcommact,
    b,constat )))


This is the longest pattern discovered in the current settings. It shows that the act of disagreeing is embedded in a series of constative acts and topic elaboration, indicating the continuous reaction of the speaker to the further development of the given interaction.

## 5. Summary

The present paper focused on the discovery of multimodal patterns of agreement/disagreement based on data from the HuComTech Corpus. It argues for a multimodal approach to human interaction by showing the interdependence of text, speech and gestures in communication, and shows the importance of implementing human behavioral patterns in more user friendly human-machine interaction systems. It describes the main features of the annotation of the corpus with emphasis on those classes that are mainly responsible for the expression of agreement/disagreement. After a short introduction to the basics of the research environment *Theme*, relevant behavioral patterns of various complexities discovered by *Theme* were presented.

## Author Contributions

The author confirms being the sole contributor of this work and has approved it for publication.

### Conflict of Interest Statement

The author declares that the research was conducted in the absence of any commercial or financial relationships that could be construed as a potential conflict of interest.
